# Factors affecting medical students in formulating their specialty preferences in Jordan

**DOI:** 10.1186/1472-6920-8-32

**Published:** 2008-05-23

**Authors:** Yousef Khader, Dema Al-Zoubi, Zouhair Amarin, Ahmad Alkafagei, Mohammad Khasawneh, Samar Burgan, Khalid El Salem, Mousa Omari

**Affiliations:** 1 Department of Public Health, Community Medicine and Family Medicine, Faculty of Medicine, Jordan University of Science and Technology, Irbid, Jordan; 2 Deprtment of Obstetrics and Gynecology, Faculty of Medicine, Jordan University of Science and Technology, Irbid, Jordan; 3 Department of peaediatrics, Faculty of Medicine, Jordan University of Science and Technology, Irbid, Jordan; 4 Department of Oral Medicine, Faculty of Medicine, University of Jordan, Irbid, Jordan; 5 Department of Neuroscience, Faculty of Medicine, Jordan University of Science and Technology, Irbid, Jordan

## Abstract

**Background:**

In recent years there has been a growing appreciation of the issues of career preference in medicine as it may affect student learning and academic performance. However, no such studies have been undertaken in medical schools in Jordan. Therefore, we carried out this study to investigate the career preferences of medical students at Jordan University of Science and Technology and determine factors that might influence their career decisions.

**Methods:**

A cross-sectional questionnaire-based survey was carried out among second, fourth and sixth year medical students at the Jordan University of Science and Technology, Irbid, Jordan during the academic year 2006/2007. A total of 440 students answered the questionnaire which covered demographic characteristics, specialty preferences, and the factors that influenced these career preferences. Possible influences were selected on the basis of a literature review and discussions with groups of medical students and physicians. Students were asked to consider 14 specialty options and select the most preferred career preference.

**Results:**

The most preferred specialty expressed by male students was surgery, followed by internal medicine and orthopaedics, while the specialty most preferred by female students was obstetrics and gynaecology, followed by pediatrics and surgery. Students showed little interest in orthopedics, ophthalmology, and dermatology. While 3.1% of females expressed interest in anesthesiology, no male students did. Other specialties were less attractive to most students.

Intellectual content of the specialty and the individual's competencies were the most influential on their preference of specialty. Other influential factors were the "reputation of the specialty", "anticipated income", and "focus on urgent care".

**Conclusion:**

Surgery, internal medicine, pediatrics, and obstetrics and gynaecology were the most preferred specialty preferences of medical students at Jordan University of Science and Technology.

## Background

Medical education requires undergraduate students to study a wide range of medical specialties. It is often assumed that students do not make their career preferences until after they have graduated from medical school. However, not only medical school entrants [1], but even medical school applicants, often have strong preferences for or against some medical careers [2-4]. Much research has concentrated on the personal characteristics of individuals choosing particular careers [5,6], on background factors in childhood influencing career preference [7,8], and on associations with particular personality types [9]. Other research has concentrated on the careers of specific groups, such as women doctors [9], on attitudes towards specific specialties, such as psychiatry [10] and anesthesia [11,12], and on the basic statistics necessary for workforce planning [13].

With the continuing evolution of health care delivery and with advances in medical technology, the appropriate specialty mix within the medical workforce is still debated. Studying career preference can help provide important information to aid in planning educational programs, set priorities, and plan for the provision of adequate health care. The preference of medical specialties chosen by medical graduates plays an important part in the future workforce in health-care system, especially in times of over or undersupply of doctors.

There are four medical schools in Jordan with the same six-year program. The first year covers general sciences. The second and third years introduce the basic biomedical sciences in a modular format. The fourth and fifth years cover clinical clerkships, in which the clinical specialties of surgery, medicine, pediatric, and obstetrics and gynecology are introduced along with selected subspecialties such as radiology, anesthesia, dermatology and ophthalmology. During the sixth year, clerkships in the four major specialties are repeated, with the option of having two elective months. The six years are equivalent to 256 credit hours, each costing an average of $100. The number of students per annum is about 250. However, there is little understanding of how different medical specialties are perceived or how career preferences are made by medical students. Therefore, this study was conducted to record career preferences of medical students at Jordan University of Science and Technology and investigate factors that might influence these career decisions.

## Methods

### Setting and participants

A cross-sectional study was conducted at Jordan University of Science and Technology among the second, fourth and sixth year medical students. The questionnaire was administered to 205 of the 418 second year students, 191 of the 250 fourth year students, and 166 of the 185 sixth year students in the medical course. The students were selected on the basis of their availability in groups at a single site. All of the selected students received a copy of the questionnaire which assessed demographics and specialty preferences. The students were handed the questionnaires by the study team and were asked to return the completed questionnaires to lecture hall attendants, the public health department or members of the study team. Of 562 questionnaires, 440 (77.7%) were returned. Response rates among second, fourth and sixth year medical students were 83.9%, 70.7%, 80.1% respectively.

### Data Collection

The questionnaire covered demographic characteristics, specialty preferences, and factors that influenced career preferences. Possible influences were selected on the basis of literature reviews and discussions with medical students and physicians. Demographic characteristics included the student's age, gender, religion, nationality, the population of community where the student completed high school, total family income, father's and mother's age, education and occupation, total number of family members, birth order and means of university financial support.

Students were asked to consider 14 specialty options and select the most preferred career preference. Specialties listed were surgery, obstetrics and gynaecology, paediatrics, internal medicine, psychiatry, orthopaedics, ophthalmology, dermatology, anaesthesiology, radiology, public health, family medicine, basic sciences, and ear, nose, and throat (ENT).

In the third part of the questionnaire influences on specialty preferences were indicated in order to assess the factors to which students attach importance when choosing their first specialty preferences. Responses to the influences were categorized as minor or major influences. These influences included hours of practice, on-call schedule, flexibility of specialty, interaction with other physicians, the reputation of the specialty, the duration of the residency program, work pressure, interest in research, interest in long term relations with patients, physician-patient interaction, diversity of patients, anticipated income, focus on community health, focus on urgent care, the curriculum, the intellectual content of specialty, the individual's competencies, mentor emulation, advice from faculty members, advice from friends, advice from parents, and advice from practicing physicians. These influences were selected after reviewing related literature and conducting discussions with medical students and physicians.

### Data analysis

The Statistical Package for Social Sciences software (SPSS, version 11.5) was used to analyze the data. Differences in means were analyzed using independent sample t-test. Differences in proportions were analyzed using Chi-square test. A P-value of less than 0.05 was considered statistically significant.

## Results

### Basic demographics

The mean (± SD) age of the respondents was 21.1 ± 2.0 years. About one third (36%) of the respondents were female. Only 23 students were Christians. About two thirds (71%) of the respondents were Jordanians. The mean age of respondents' fathers was 52.4 ± 11.9 years and of respondents' mothers was 46.1 ± 9.1 years. A total of 75 (17%) students had a physician father, 23 students had a physician mother, and 110 of them had at least one brother or sister who studied medicine. Other socio-demographic characteristics and school related variables of the respondents are shown in Table 1.

**Table 1:** The socio-demographic characteristics and school related variables of all respondents.

	Male (n = 280) n (%)	Female (n = 160) N (%)	Total (N = 440) n (%)	p-value*
Level				0.890
Second year	109 (38.9)	63 (39.4)	172 (39.1)	
Fourth year	88 (31.4)	47 (29.4)	135 (30.7)	
Sixth year	83 (29.6)	50 (31.3)	133 (30.2)	
				
Nationality				0.403
Jordanian	196 (70.0)	118 (73.8)	314 (71.4)	
Others	84 (30.0)	42 (26.3)	126 (28.6)	
				
Country of the high school				0.278
Jordan	162 (57.9)	101 (63.1)	263 (59.8)	
Others	118 (42.1)	59 (36.9)	177 (40.2)	
				
Payment system				0.037
Regular	125 (44.6)	88 (55.0)	213 (48.4)	
International	155 (55.4)	72 (45.0)	227 (51.6)	
				
Total family income (JD)				0.273
500 or less	45 (16.1)	25 (15.6)	70 (15.9)	
501–1000	76 (27.1)	53 (33.1)	129 (29.3)	
1001–1500	56 (20.0)	32 (20.0)	88 (20.0)	
1501–2000	40 (14.3)	27 (16.9)	67 (15.2)	
More than 2000	63 (22.5)	23 (14.4)	86 (19.5)	
				
Father's education				0.056
Less than high school	29 (10.4)	11 (6.9)	40 (9.1)	
High school	31 (11.1)	22 (13.8)	53 (12.0)	
Diploma	17 (6.1)	13 (8.1)	30 (6.8)	
University	105 (37.5)	63 (39.4)	168 (38.2)	
Higher university degree	98 (35.0)	51 (31.9)	149 (33.9)	
				
Mother's education				0.057
Less than high school	54 (19.3)	23 (14.4)	77 (17.5)	
High school	70 (25.0)	36 (22.5)	106 (24.1)	
Diploma	53 (18.9)	36 (22.5)	89 (20.2)	
University	83 (29.6)	41 25.6)	124 (28.2)	
Higher university degree	20 (7.1)	24 (15.0)	44 (10.0)	

* Chi-square test for the difference between males and females

### Specialty preferences

The most preferred specialty among male students was surgery, followed by internal medicine and orthopaedics, while that most preferred by female students was obstetrics and gynaecology followed by pediatrics and surgery (Table 1). A total of 146 (52%) of male students expressed an interest in surgery compared to 15% of female students (p < 0.005). Gynecology was preferred by 31% of female students compared to 1% of male students. Males (15%) and females (14%) were equally likely to express interest in internal medicine. Students showed little interest in other specialties including orthopedics (8% male vs. 3% female), ophthalmology (6% male vs. 9% female), and dermatology (1% male vs. 4% female). While 3% of females expressed interest in anesthesiology, no male students did (Table 2). Other specialties including radiology, psychiatry, public health, family medicine, basic sciences, and ENT were not preferred by almost all students.

**Table 2:** Specialty preferences among 2nd, 4th, and 5th year medical students from different years of entry during the academic year 2006/2007 at Jordan University of Science and Technology according to gender and level.

	Male			Female		
		
	2^nd ^year (n = 109) n (%)	4^th ^year (n = 88) n (%)	6^th ^year (n = 83) n (%)	2^nd ^year (n = 63) n (%)	4^th ^year (n = 47) n (%)	6^th ^year (n = 50) n (%)
Surgery	70 (64.2)	48 (54.5)	28 (33.7)	16 (25.4)	4 (8.5)	4 (8.0)
Gynecology	1 (0.9)	0 (0.0)	1 (1.2)	11 (17.5)	10 (21.3)	14 (28.0)
Pediatrics	2 (1.8)	6 (6.8)	12 (14.5)	9 (14.3)	5 (10.6)	14 (28.0)
Internal medicine	9 (8.3)	18 (20.5)	14 (16.9)	2 (3.2)	12 (25.5)	8 (16.0)
Psychiatry	1 (0.9)	2 (2.3)	1 (1.2)	1 (1.6)	1 (2.1)	1 (2.0)
Orthopedics	8 (7.3)	4 (4.5)	11 (13.3)	4 (6.3)	0 (0.0)	0 (0.0)
Ophthalmology	5 (4.6)	4 (4.5)	7 (8.4)	6 (9.5)	7 (14.9)	2 (4.0)
Dermatology	2 (1.8)	0 (0.0)	2 (2.4)	2 (3.2)	1 (2.1)	4 (8.0)
Anesthesia	0 (0.0)	0 (0.0)	0 (0.0)	5 (7.9)	0 (0.0)	0 (0.0)
Radiology	0 (0.0)	1 (1.1)	2 (2.4)	0 (0.0)	1 (2.1)	1 (2.0)
Ear, nose, and throat	2 (1.8)	0 (0.0)	2 (2.4)	1 (1.6)	0 (0.0)	0 (0.0)
Public health	2 (1.8)	0 (0.0)	0 (0.0)	0 (0.0)	2 (4.3)	1 (2.0)
Family medicine	0 (0.0)	1 (1.1)	2 (2.4)	1 (1.6)	2 (4.3)	0 (0.0)
Basic science	0 (0.0)	3 (3.4)	0 (0.0)	2 (3.2)	1 (2.1)	1 (2.0)
Others	7 (6.4)	1 (1.1)	1 (1.2)	3 (4.8)	1 (2.1)	0 (0.0)

**Figure 1:**
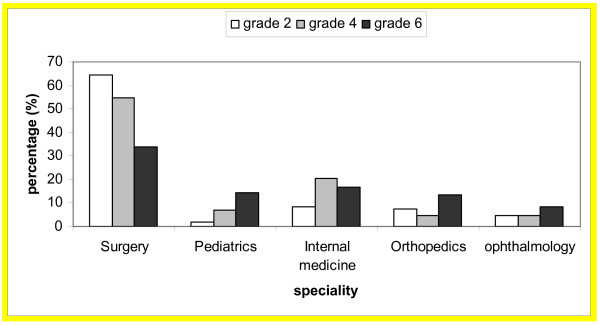
The five most attractive specialties for male medical students at Jordan University of Science and Technology by level.

**Figure 2:**
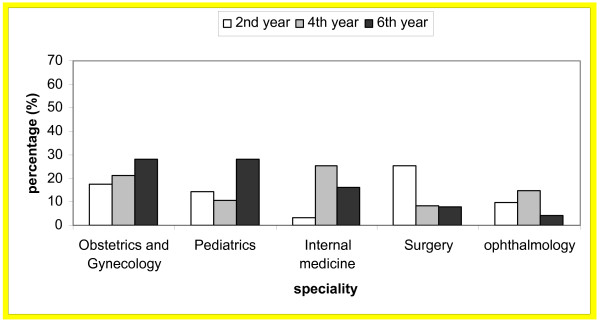
The five most attractive specialties for female medical students at Jordan University of Science and Technology by level.

Specialty preferences among medical students by gender and level are shown in Table 2. The changes in preference of the five most attractive specialties by level for males and females are shown in Figures 1 and 2. Interest in surgery decreased as students advanced through the course among both males and females while interest in pediatrics increased among males only. Interest in internal medicine was the lowest for second year medical students and highest for fourth year medical students.

### Factors that influence specialty preference

Of the total, 84% of respondents rated "intellectual content of the specialty", 64% rated "individual's competencies" as influential on their preference of specialty (Table 3). Other important factors rated as influential were "reputation of the specialty" (59%), "anticipated income" (58%) and "focus on urgent care" (55%). Less common variables influencing career preference were physician-patient interaction, advice from faculty or friends, and on-call schedule.

**Table 3:** Factors influencing specialty preferences among medical students at Jordan University of Science and Technology according to gender.

	Male (n = 280) n (%)	Female (n = 160) n (%)	Total N (%)	P-value *
Hours of practice	41 (14.6)	34 (21.3)	75 (17.0)	0.076
On-call schedule	29 (10.4)	32 (20.0)	61 (13.9)	0.005
Flexibility of specialty	127 (45.4)	57 (35.6)	184 (41.8)	0.046
Interaction with physicians	132 (47.1)	67 (41.9)	199 (45.2)	0.286
Specialty reputation	183 (65.4)	76 (47.5)	259 (58.9)	0.000
Duration of residency program	60 (21.4)	41 (25.6)	101 (23.0)	0.314
Work pressure	71 (25.4)	34 (21.3)	105 (23.9)	0.331
Interest in research	118 (42.1)	53 (33.1)	171 (38.9)	0.062
Interest in long term relations with patients	96 (34.3)	62 (38.8)	158 (35.9)	0.348
Physician-patient interaction	32 (11.4)	17 (10.6)	49 (11.1)	0.797
Diversity of patients	131 (46.8)	72 (45.0)	203 (46.1)	0.718
Anticipated income	183 (65.4)	72 (45.0)	255 (58.0)	0.000
Focus on community health	78 (27.9)	72 (45.0)	150 (34.1)	0.000
Focus on urgent care	163 (58.2)	78 (48.8)	241 (54.8)	0.055
Curriculum	102 (36.4)	68 (42.5)	170 (38.6)	0.208
Intellectual content of the specialty	239 (85.4)	132 (82.5)	371 (84.3)	0.428
Individual's competencies	185 (66.1)	96 (60.0)	281 (63.9)	0.202
Emulate a physician	119 (42.5)	65 (40.6)	184 (41.8)	0.701
Advice from faculty	31 (11.1)	18 (11.3)	49 (11.1)	0.954
Advice from friends	36 (12.9)	13 (8.1)	49 (11.1)	0.129
Advice from parents	93 (33.2)	60 (37.5)	153 (34.8)	0.364
Advice from practicing physicians	73 (26.1)	45 (28.1)	118 (26.8)	0.640

* Chi-square test for the difference between males and females

Factors of particular importance to female students compared with their male counterparts were "on-call schedule" (p < 0.005) and focus on community health (p < 0.0005). In contrast, flexibility of specialty (p < 0.046), specialty reputation (p < 0.0005), and anticipated income (p < 0.0005) were more influential for male compared to female students.

## Discussion

The career preferences made by medical students and doctors and factors influencing these preferences are of importance to medical workforce planners especially in times of oversupply or undersupply of doctors. Surgery, internal medicine, pediatrics, and obstetrics and gynaecology were the most specialty preferred preferences among medical students at Jordan University of Science and Technology. The findings of this study were similar to these reported in other studies [14,15].

Gender differences were noted in the preference of certain specialties. Female students preferred pediatrics and obstetrics and gynaecology, but were less likely to choose surgery. This finding is in agreement with others [9,16,17]. It was reported that female students seemed to have a more idealistic approach than male students, and were less often influenced by the prospect of a good income or prestige.

Surprisingly, it was noticed that no male students planned for a career in anesthesia. It perhaps related to an increase in the number of nurse anesthetists employed at university hospitals, suggesting that career opportunities might be diminished.

Family medicine was one of the least popular specialty preferences, which is consistent with the findings of other studies [18,19]. However, having some students doing an ambulatory off site rotation at the time of the study could have resulted in sample selection bias, as these students, who might have been more interested in primary care medicine, would have missed the opportunity to participate. We believe that this effect is very small as most of our students doing off site ambulatory rotation do so as part of their "compulsory" curriculum and not as an elective.

Although this survey did not follow up students, it was noticed that the distribution of students' first specialty preference often changed as they progressed through the course. Second year medical students' preferences were inclined towards surgery with this specialty chosen as a first career preference by more than a half of the group surveyed. A wider distribution of first specialty preference was found among fourth year medical students but the widest was found among sixth year medical students. Only 24% of sixth year medical students were interested in surgery. As second year medical students still did not have clinical experience, the majority of them tended to choose surgery, perhaps because of its perceived prestigious status among medical specialties. In contrast, sixth year medical students were more evenly spread in their preferences of specialty fields, which were less based on their social perceptions.

Many researchers have tried to determine factors that influence students' specialty preferences [5,6,20]. Some have postulated that the primary influences were students' personal characteristics, such as controllable lifestyles whereas others have suggested other factors relating to medical school characteristics such as orientation toward research. Medical educators, however, have focused on educational influences such as curriculum, primary care experiences, and faculty role models. These influences are more readily modifiable than are such factors such as institution's relative research intensity or students' long-held values.

Surgery may lend itself more readily to the influence of role models. A surgeon who serves as a role model may be a parent or other relative, neighbor, personal physician, family friend, or medical school faculty members. Regardless of the source, role models were a powerful influence on the career preference of 41.8% of students in this study, with the majority being male students who preferred surgery. General surgery and its derivative specialties continue to appeal to graduating students in many countries [22,23].

Several studies have cited clinical role models as being important influences on students' residency preferences [5,21-24]. This included negative role models, who drove students away from some specialties [23]. In addition to faculty, resident role models have been occasionally cited as influential [21,22]. However, for the most part, those studies have been retrospective, so it was difficult to determine whether their data arose from actual influence or from students' fond memories. One prospective study noted that students exposed to general internists as role models in their internal medicine clerkship were more likely to choose primary care careers, but the study measured this with a post-clerkship survey and did not determine actual residency preferences [24]. Much interest has been focused on factors influencing preference of career in primary care. Countries with strong primary care system have better health outcomes, fewer unnecessary deaths and lower costs than those with poor primary care [25]. There is therefore an incentive for ensuring adequate recruitment into primary care.

It is likely that a balance of factors operating before, during and after medical school is involved in any individual's career decision. Future studies should consider the influences on the specialty preference of students from many institutions, across several clerkships, and over many years. Further research is clearly needed to identify which unidentified factors impact on graduates' career preferences and which of these can be manipulated to influence career preferences in a particular direction, bearing in mind that influencing career preference in one direction may have unpredictable and unwanted effects on preferences in another direction

## Conclusion

The most preferred specialty preferences of medical students at Jordan University of Science and Technology Surgery were internal medicine, pediatrics, and obstetrics and gynaecology. Future studies should consider the influences on the specialty preference of students from other institutions, across several clerkships, and over the early years of clinical practice.

## Abbreviations

ENT: ear, nose and throat.

## Competing interests

The authors declare that they have no competing interests.

## Authors' contributions

YK and DA–Z participated in the design of the study and coordinated its implementation, and performed the statistical analysis. ZA, AK, MK, SB, KES and MO participated in the study design and made essential contributions to the different versions of the manuscript. All authors read and approved the manuscript.

## Pre-publication history

The pre-publication history for this paper can be accessed here:


